# Experiences from Clinical Research and Routine Use of Florbetaben Amyloid PET—A Decade of Post-Authorization Insights

**DOI:** 10.3390/ph17121648

**Published:** 2024-12-07

**Authors:** Aleksandar Jovalekic, Santiago Bullich, Núria Roé-Vellvé, Guilherme Domingues Kolinger, Lorelei R. Howard, Floriana Elsholz, Mariana Lagos-Quintana, Beatriz Blanco-Rodriguez, Esther Pérez-Martínez, Rossella Gismondi, Audrey Perrotin, Marianne Chapleau, Richard Keegan, Andre Mueller, Andrew W. Stephens, Norman Koglin

**Affiliations:** 1 Life Molecular Imaging GmbH, Tegeler Str. 7, 13353 Berlin, Germany; 2 Life Molecular Imaging Inc., 75 State Street, Floor 1, Boston, MA 02109, USA

**Keywords:** amyloid, Alzheimer’s disease, Centiloid, florbetaben, mild cognitive impairment, quantification, reimbursement

## Abstract

Florbetaben (FBB) is a radiopharmaceutical approved by the FDA and EMA in 2014 for the positron emission tomography (PET) imaging of brain amyloid deposition in patients with cognitive impairment who are being evaluated for Alzheimer’s disease (AD) or other causes of cognitive decline. Initially, the clinical adoption of FBB PET faced significant barriers, including reimbursement challenges and uncertainties regarding its integration into diagnostic clinical practice. This review examines the progress made in overcoming these obstacles and describes the concurrent evolution of the diagnostic landscape. Advances in quantification methods have further strengthened the traditional visual assessment approach. Over the past decade, compelling evidence has emerged, demonstrating that amyloid PET has a strong impact on AD diagnosis, management, and outcomes across diverse clinical scenarios, even in the absence of amyloid-targeted therapies. Amyloid PET imaging has become essential in clinical trials and the application of new AD therapeutics, particularly for confirming eligibility criteria (i.e., the presence of amyloid plaques) and monitoring biological responses to amyloid-lowering therapies. Since its approval, FBB PET has transitioned from a purely diagnostic tool aimed primarily at excluding amyloid pathology to a critical component in AD drug development, and today, it is essential in the diagnostic workup and therapy management of approved AD treatments.

## 1. Introduction

### 1.1. Background on Alzheimer’s Disease and Amyloid PET Imaging

Alzheimer’s disease (AD) is the leading cause of dementia, accounting for 60–80% of cases in individuals over the age of 65. It represents a growing public health crisis due to its increasing prevalence and the substantial burden it imposes on individuals, families, and healthcare systems [1,2]. These factors create a significant societal and financial burden, making AD one of the most emotional and costly life-threatening diseases globally [3,4,5].

AD has a protracted asymptomatic preclinical phase, during which pathological changes already occur in the brain. Next follows a period characterized by subjective or mild cognitive impairment. Early symptoms are rather non-specific, making an accurate diagnosis of AD challenging or even impossible when based on cognitive tests alone. However, AD-related pathological changes can be detected through specific biomarkers, providing an opportunity for earlier diagnosis and intervention, even at very early stages, before clinical symptoms appear. The disease is characterized by the persistent accumulation of amyloid-beta (Aβ) plaques, which eventually lead to neuroinflammation, tau-dependent neurodegeneration and synaptic dysfunction. Over time, these pathophysiological processes result in further cognitive decline and clinical symptoms, marking progression in the AD continuum until the dementia phase, which is ultimately fatal [6].

The specific detection and quantification of AD-related biomarkers is essential in current diagnostic pathways [7,8]. Among the various biomarkers, Aβ plays a central role in the diagnostic process. The only method for the direct in vivo detection of Aβ plaques as AD biomarkers is amyloid positron emission tomography (PET) imaging. This minimally invasive imaging technique uses 18F- or 11C-labeled radiotracers that selectively bind to Aβ aggregates in the brain, allowing for their visualization and quantification. This capability is particularly valuable for confirming the presence of amyloid pathology, distinguishing AD from other dementias, and guiding treatment decisions.

While AD pathology can also be measured through fluid biomarkers such as cerebrospinal fluid (CSF) or blood samples, these approaches only indirectly reflect brain amyloid status by capturing soluble fragments, which are dependent on current protein production and clearance rates, providing a snapshot of the pathologic state at a given point in time [9]. In contrast, amyloid PET imaging allows for the direct visualization of amyloid plaques that have accumulated over time, sometimes over decades, thus representing the full magnitude and localization of neuropathologic load. Furthermore, recent findings suggest that amyloid PET may detect Aβ deposition earlier than CSF biomarkers, potentially allowing for earlier intervention [10].

Florbetaben (FBB) is an established and globally used radiopharmaceutical for the PET imaging of amyloid deposition in the brain. The development and adoption of this imaging biomarker align with the growing understanding of and advancements in the diagnostic landscape of AD [7,8]. In this article, we will discuss how the introduction of new biomarkers, such as amyloid PET, has transformed the AD diagnostic process, shifting from a primarily clinical, late-stage approach to a more biomarker-driven framework, facilitating the earlier diagnosis of AD even before clinical symptoms appear.

The clinical development of FBB will be briefly summarized, focusing on the pivotal Phase 3 study that demonstrated its diagnostic efficacy in an autopsy-verified cohort [11]. The validated and approved visual assessment procedure for FBB PET image interpretation will be reviewed [12]. Recent real-world image read data from a post-authorization safety study (PASS1, EUPAS12145) will also be highlighted, underscoring the feasibility of routine practice [13]. Additionally, the adjunct use of quantitative methods for PET scan analysis will be discussed, paying particular attention to the Centiloid scale [14] and its further validation and growing adoption [15,16].

Furthermore, the clinical utility of amyloid PET will be explored through data from large, established real-world cohorts such as IDEAS and ABIDE, as well as data from memory clinics where FBB PET was integrated seamlessly into routine clinical practice [17,18,19]. Insights from meta-analyses [20] and findings from a post-authorization safety study (PASS2, EUPAS13366) will be discussed to further validate its real-world impact [13].

Additionally, the important role of amyloid PET, including FBB, in disease-modifying drug (DMD) trials will be emphasized, as it has been instrumental in facilitating the development of new therapies by confirming the presence of the therapeutic target, reducing misdiagnoses, and enabling the monitoring of amyloid clearance during treatment. Lastly, we will offer an outlook on the role of FBB PET in the evolving landscape of AD diagnostics and therapies.

### 1.2. Evolution of the AD Diagnostic Landscape: From a Phenotype-Based to Biomarker-Assisted Diagnosis

Before the introduction and validation of molecular imaging with specific PET radiotracers for the detection of Aβ in AD patients, a definitive diagnosis of AD could only be confirmed post-mortem through autopsy and histopathological evaluation [21,22]. During this time, a clinical diagnosis of AD was based on a set of criteria fully dependent on the patient’s cognitive and functional decline [21]. Autopsy studies, however, revealed a significant rate of misdiagnosis when relying solely on clinical criteria, with error rates ranging from 20% to 30% at advanced stages of the disease, and with even higher error rates at early disease stages [22].

**Figure 1:**
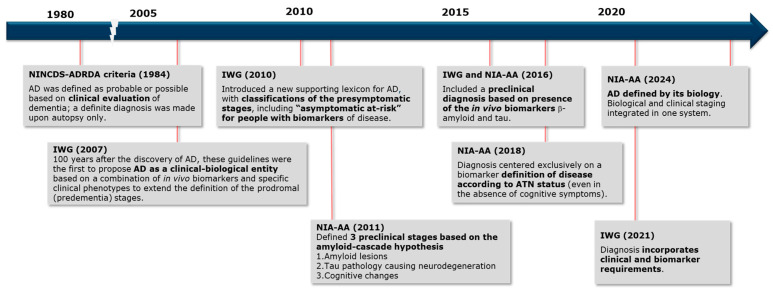
Development of Diagnostic Criteria for Alzheimer’s Disease. Key milestones in the evolution of diagnostic criteria are outlined. All updates since 2007 introduce biological criteria in the classification of AD, in addition to clinical symptoms [7,8,9,21,23,24,25,26,27].

Recognizing the limitations of relying purely on clinical diagnosis, several updates to the definition of AD have been proposed since 2007 by the International Working Group (IWG) [8,23,24,25,26] and the National Institute on Aging and Alzheimer’s Association (NIA-AA) [7,9,21,27] (see Figure 1). These updates have progressively emphasized the need for biological evidence alongside clinical assessment. This shift in diagnostic guidelines reflects the growing understanding that AD, when defined by clinical phenotype alone, may not fully capture the underlying pathological processes [22]. In parallel, advances in diagnostic biomarkers and both imaging and fluid-based tests have allowed for the measurement of AD-related pathologies, significantly improving diagnostic accuracy [28].

### 1.3. Molecular Biomarkers for Improving Diagnosis and Enabling Drug Development

The accurate and early diagnosis of AD relies on detecting the core pathophysiological biomarkers of the disease—amyloid-beta (A), tau (T), and neurodegeneration (N) [9,27], although additional biomarkers are also emerging [7]. These core biomarkers of AD pathology, often referred to as the “A/T/N” framework [27], can be assessed using various techniques, including PET, CSF analysis, and, more recently, blood-based assays.

PET imaging with specific tracers allows for the in vivo visualization and quantification of underlying pathologies, such as amyloid and tau deposition, providing spatial information on the distribution of these pathologies within the brain. Unlike fluid biomarkers, the ability to capture the magnitude and topographical detail of the pathology offered through PET imaging enables the definition of biological stages of the disease process. While individual fluid biomarkers can indicate that an individual has entered a biological stage of AD and may also provide additional information on staging after further validation, they currently lack the ability to differentiate between specific stages along the disease continuum, as defined in the revised criteria for diagnosis and staging of AD in 2024 [7]. Fluid biomarkers quantify the concentration of proteins collected in CSF or blood and provide a temporary snapshot of the pathological processes occurring at a specific point in time. These markers reflect both the production and clearance rates of proteins via the measurement of soluble components of amyloid pathology, thus only offering a temporary glimpse into the current disease state. In contrast, amyloid PET imaging allows for the visualization of amyloid plaques that have built up over time, giving a more comprehensive view of the extent and localization of neuropathological changes. Recent findings suggest that amyloid PET may detect Aβ deposition earlier than CSF biomarkers [10]. Lowe et al. (2024) used highly accurate Aβ CSF measurements and optimized Aβ PET methods designed to detect early Aβ deposition. The study found that Aβ PET showed an apparent earlier detection of Aβ accumulation than CSF p-tau181/Aβ42 or Aβ42/40 ratios in cross-sectional data, with autopsy confirmation in a subset of participants [10].

Cerebrospinal fluid biomarkers, while able to assess multiple markers from a single sample, require an invasive lumbar puncture and do not provide spatial information about the location of amyloid or tau pathology in the brain. Blood-based biomarkers are an emerging area of research and hold promise due to their minimally invasive nature, relatively lower cost, and higher accessibility, making them more suitable for screening purposes and use even at the general practitioner level. This may streamline the referral process and initial diagnostic workup. While blood assays can detect various biomarkers, they also lack the ability to directly localize and quantify the pathology within the brain. Although fluid biomarkers show great promise and may help guide the use of more advanced techniques like amyloid PET, significant limitations—such as interference from comorbidities [29] and medications [30]—still need to be addressed [31].

Large efforts in the pharmaceutical industry have focused on the development of amyloid plaque-removing therapies, where molecular biomarkers played a crucial role in selecting the optimal dose [32], in the verification of eligibility criteria for enrollment into clinical studies (i.e., confirming the presence of brain amyloid plaques), for monitoring amyloid removal [33,34] and for treatment discontinuation decisions [35,36].

## 2. Overview of the Florbetaben Development Program

Moreover, 18F-FBB is a radiopharmaceutical that selectively binds to Aβ plaques in the brain. Chemically, it is an 18F-labeled polyethylene glycol stilbene derivative. When the 18F radioisotope decays, a positron is emitted. This positron interacts with a nearby electron in the surrounding tissue/cells, resulting in an annihilation and the release of two photons in the same line and in opposite directions. These pairs of photons are then detected by a PET scanner, allowing for the precise localization and quantification of the PET signal. The 18F radioisotope has a radioactive decay half-life of approximately 110 min, making it more compatible for routine use, compared to 11C-labeled PET tracers, such as 11C-PIB, which have a much shorter half-life of approximately 20 min. The centralized production of 18F-labeled radiopharmaceuticals allows for remote distribution to multiple PET imaging sites. The delivery logistics are well-established from the distribution of 18F-FDG—the most widely used PET tracer—and were expanded to other 18F-labeled radiopharmaceuticals.

**Figure 2:**
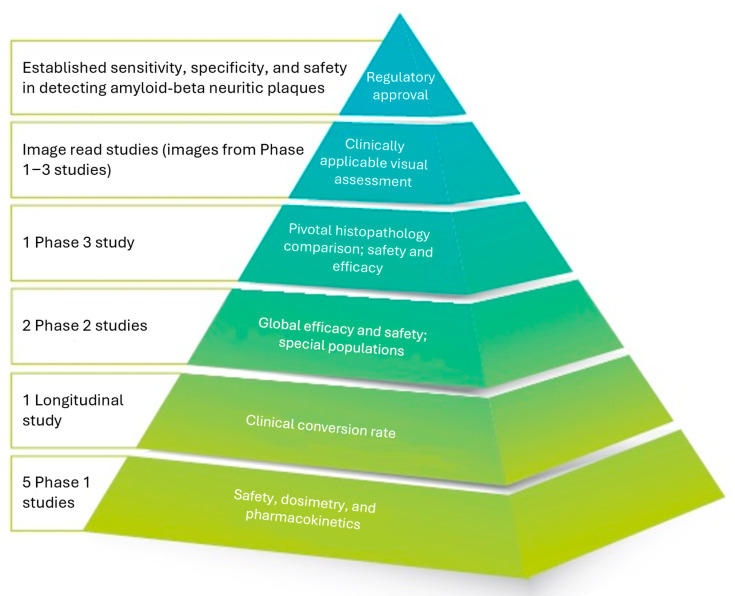
The regulatory approval of florbetaben for use in clinical practice was supported by a robust clinical development program. This program validated the diagnostic accuracy and safety of florbetaben through a series of pivotal studies, establishing its role in Alzheimer’s diagnostics.

The FBB molecule was first described by Zhang et al. in 2005 [37], who developed a series of compounds and tested them in both in vitro and in vivo models. The rights to the lead compound were licensed by the German pharmaceutical company Schering AG, which was later acquired by Bayer AG, who then led the clinical development program. The general suitability of FBB binding to amyloid-beta plaques in the brain of AD patients and for PET imaging was then verified in a first in-human study [38]. With this, florbetaben was the first 18F-labeled amyloid PET tracer investigated in humans. In 2012, the rights to florbetaben were taken over by the spin-off company Piramal Imaging, which completed the clinical development program and obtained marketing authorizations in the EU and US. In total, the development program encompassed 1090 administrations in 872 subjects across nine clinical trials and two non-interventional image-reading studies, establishing a robust safety and efficacy profile (see Figure 2, [39]). Ownership of the product changed again when Piramal Imaging was acquired by the Life Healthcare Group, resulting in a name change of the company to Life Molecular Imaging. Regardless of the changes in owners and company names, the company’s core functions for research, clinical development, chemistry, manufacturing and control, regulation and quality remained with the same team in Berlin (Germany).

Several Phase 1 studies have evaluated the pharmacokinetic and pharmacodynamic properties of florbetaben (FBB), providing proof-of-concept in both patients and healthy volunteers [40]. The prognostic accuracy of FBB was investigated in a prospective, longitudinal study involving 45 patients with MCI [41,42].

At baseline, 24 (53%) of the MCI patients had positive FBB PET scans. After 48 months, 21 individuals (87.5%) of these PET-positive patients progressed to AD dementia, compared to 0 (0%) of the 21 PET-negative patients. Five individuals (24%) who were PET-negative were diagnosed with non-AD dementia at 48 months. These conversion rates resulted in a predictive accuracy of 94% (95% CI 74% to 99%) [41,42].

A Phase 2 study, using a clinical dementia diagnosis as the reference standard, demonstrated FBB’s potential to differentiate between AD and non-AD patients, laying the foundation for the image reading methodology [43,44].

A key component of the FBB development program was a global Phase 3 histopathology study that involved brain PET imaging of end-of-life individuals who had consented to brain donation post-mortem to verify that the PET tracer had truly bound to Aβ plaques. Among these participants, 74 brains were assessed for the presence of neuritic Aβ plaques [11]. The visual assessment of FBB PET scans demonstrated high sensitivity and specificity in detecting these plaques. Out of 47 subjects with confirmed β-amyloid pathology, 46 were correctly identified as positive through the visual interpretation of their PET scans, yielding a sensitivity of 97.9%. Conversely, scans from 24 out of 27 subjects without β-amyloid were accurately classified as negative, resulting in a specificity of 88.9% [11].

These findings demonstrated that positive scans provide critical information for determining whether cognitive impairment is associated with Aβ pathology, such as that present in AD. Negative scans, on the other hand, allow clinicians to explore alternative causes of cognitive decline that are unrelated to Aβ deposition. The study also yielded high predictive values for the absence or presence of Aβ pathology, with a negative predictive value (NPV) at 96.0% and a positive predictive value (PPV) at 93.9%.

Moreover, when exactly matching the PET scan with specified tissue regions, the study established a strong correlation between the FBB signal in PET imaging and the extent of Aβ deposition in the histopathological analysis of autopsied brains. Both visual and quantitative assessments showed largely overlapping results [11].

In all developmental studies, FBB was well-tolerated, with no serious adverse events (AEs) related to the drug. The most common AEs reported were related to the injection site, such as pain, erythema, and irritation [39,42]. The extensive clinical development program, particularly the pivotal Phase 3 study and the positive risk-benefit assessment, led to the approval of FBB by the European Medicines Agency (EMA) and the U.S. Food and Drug Administration (FDA) in 2014 [11,40] and to the start of marketing it under the brand name Neuraceq^®^. Since then, marketing authorizations have been obtained from several other national regulatory bodies worldwide. Neuraceq^®^ is marketed by LMI in the EU, the UK and the US, and by license partners in Canada, China, Japan, South Korea, Switzerland and Taiwan. In Australia, Brazil, Chile, New Zealand, Thailand, and the Philippines, FBB is supplied according to local regulations and/or exemptions to the requirement for marketing authorizations.

## 3. Florbetaben: Visual Assessment and Quantification in Clinical Settings

### 3.1. Visual Assessment

The method for visual interpretation was validated through comparisons with histopathology data in an end-of-life population [11,12]. Positive FBB PET scans correspond to moderate or frequent amounts of amyloid plaques, while negative scans reflect no or a sparse amyloid plaque burden [39,42]. Regulatory agencies required further validation of the visual assessment method prior to approval. Therefore, additional image-reading studies were performed, which demonstrated the high reproducibility and consistency of the visual assessment method. These studies confirmed that visual interpretation was equally effective, irrespective of whether the readers received electronic or dedicated in-person training or were naïve or experienced interpreters [12]. These findings were further supported by studies conducted with international readers, achieving high concordance across geographical regions. A non-interventional read study (Japan-Read study) reported a 100% concordance between the majority of reads obtained by readers trained in English and Japanese languages and high inter-reader reliability for the Japanese-trained readers (Fleiss’ k = 0.87 (95% CI: 0.78–0.96) [45]. This validation further established visual assessment as the standard method for clinical practice [12].

In clinical settings, FBB PET imaging has gained increasing use for supporting the diagnosis of AD [18]. The PET images are interpreted using a dichotomous visual readout, determining the presence or absence of β-amyloid neuritic plaques in the brain, i.e., an amyloid-positive or amyloid-negative scan [39,42].

**Figure 3:**
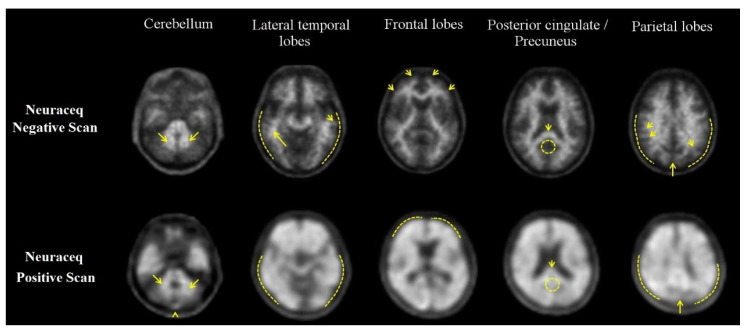
Visual patterns of florbetaben PET scans—axial views of negative (**top row**) and positive (**bottom row**) PET scans. Key features include tracer uptake in cortical regions, variations in gray and white matter contrast, and distinct anatomical markers. In the cerebellum, white matter contrast (arrows) is visible in both negative and positive scans, with extracerebral tracer uptake in the scalp and posterior sagittal sinus (arrowhead). In the lateral temporal lobes, the positive scan shows a smooth, rounded outer brain border (dashed line) due to gray matter uptake, while the negative scan has a more jagged, mountainous white matter pattern (arrows). In the frontal lobes, tracer uptake in positive scans results in a smooth appearance (dashed line), compared to the spiculated white matter seen in negative scans (arrows). In the posterior cingulate/precuneus, a negative scan reveals a hypointense region adjacent to the splenium (circle), which is absent in positive scans. Lastly, in the parietal lobes, positive scans display a thinner midline and smoother cortical areas, with tracer uptake extending to the outer edge, while in negative scans, the midline is clearly delineated (long arrow) and white matter has a spiculated appearance (short arrow), with less uptake at the cortical rim (dashed line) (from [39]).

For visual assessment, interpretation is based on tracer uptake in cortical gray matter relative to the adjacent white matter. Key regions evaluated include the temporal, frontal, and parietal lobes, as well as the posterior cingulate cortex/precuneus [39,42]. Figure 3 illustrates these visual patterns, highlighting both positive and negative scan features.

### 3.2. Quantification

Quantitative amyloid PET measures have been used in research since the introduction of the 11C-labeled Pittsburgh compound B (PiB) in 2004 [46]. Quantification, unlike binary visual reads, offers a continuous measure of amyloid burden, helping to capture nuanced differences in amyloid deposition and localization [47]. FBB PET has been shown to enable the early detection of amyloid load [48] and to detect longitudinal changes that are only measurable through quantitative methods [49].

The standardized uptake value ratio (SUVR) approach is the most used metric, representing the ratio of tracer uptake between target and reference regions during a static PET acquisition [47]. Its accuracy is dependent on factors like tracer, reference region, and delineation method, limiting the comparability of SUVR data across studies [47]. To address the need for harmonization across different tracers and PET centers, the Centiloid scale was developed, which is also based on SUVR analysis [14]. This metric allows for a standardized comparison of amyloid burden across imaging centers, tracers, and analytical platforms. On the Centiloid scale, 0 CL represents the mean gray matter signal of young healthy controls, while 100 CL reflects the average amyloid load in typical mild AD patients [14]. This method also harmonizes what brain regions should be considered target regions and reference regions. Various studies have explored optimal CL thresholds for distinguishing amyloid-negative from amyloid-positive scans [16]. Such thresholds can be derived from histopathology samples, where distinguishing between none-to-sparse and moderate-to-frequent amyloid plaque density yields a range of optimal CL thresholds [16]. Conservative analysis suggests a cutoff of approximately 35 CL [15].

**Figure 4:**
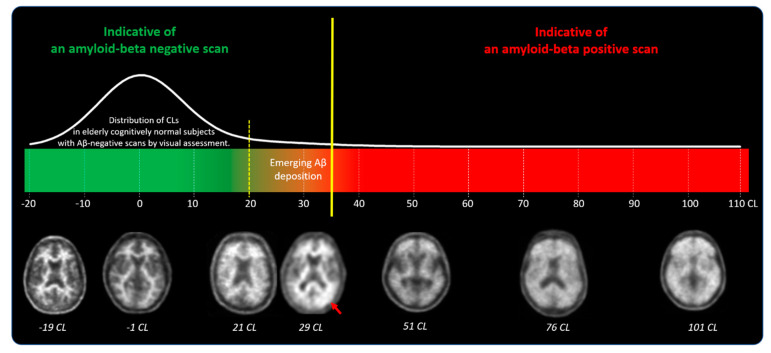
Illustration of how quantitative information can supplement visual assessment of FBB PET scans. Centiloid values above 35 CL indicate established Aβ accumulation corresponding to a density of moderate and frequent neuritic plaques by neuropathology. Centiloid values below 20 represent elderly cognitively normal subjects with visually negative amyloid-beta scans. Centiloid values in the range between 20 and 35 CL are more likely to be ambiguous, can be either negative or positive by visual assessment, and often correspond to subjects with emerging Aβ deposition. Readers should carefully examine such scans to detect subtle amyloid accumulation, which may present as focal or unilateral uptake (red arrow) (from [15]).

Figure 4 is an illustrative example of the Centiloid scale.

The Centiloid scale has been used extensively to improve the standardization of Aβ PET quantification across tracers, scanners, and analytical implementations [14,15,47,50,51]. Within the Amyloid Imaging to Prevent Alzheimer’s Disease (AMYPAD) initiative, the potential bias associated with Centiloid pipeline design options was evaluated using 32 different pipelines. It was shown that the Centiloid metric is robust against pipeline design options and factors, which resulted in mean marginal changes in the order of the test–retest variability (~3 CL) of the Centiloid metric [50]. The European Medicines Agency (EMA) recently issued a qualification opinion endorsing the Centiloid metric as a validated measure of global amyloid load for clinical trials when combined with appropriate quality control [52].

The quantification of FBB PET images using different metrics was explored in detail in the florbetaben label extension (fLEX) study [15]. High concordance rates between quantification and visual assessments have been reported, both in clinical trials with consensus reads (~95%, [15,53,54,55]) and in clinical real-world situations with local clinical visual reads (~85%, [56,57]). Up to ~15% of amyloid scans can yield discordant quantification and visual assessment outcomes [56,57]. These discordant cases typically present clinically as challenging cases. The added value of quantification in such cases has recently been demonstrated in a cohort of complex amyloid PET scans [58]. Additionally, several amyloid PET image analysis tools have been certified, expanding the scope for quantifying FBB PET scans in clinical settings [16,47]. In the EU and UK, the EMA and MHRA have approved the quantification of florbetaben PET images as an adjunct to the previously approved visual image interpretation methodology, with some quantification pipelines also including Centiloid analysis [42,59]. The Centiloid scale offers valuable insights for predicting disease progression [60,61]. For instance, van der Kall et al. (2021) found that moderate to high amyloid levels are associated with a more rapid rate of progression, underscoring the potential of quantitative amyloid measures as predictive tools in research contexts [60]. Quantitative measures, such as the Centiloid scale, are essential tools for accurately tracking amyloid load over time, offering valuable insights for clinical decision-making, e.g., in the efficacy assessment of amyloid-lowering drugs (see Section 5).

### 3.3. Visual Assessment in Real-World Settings and Quantification as an Adjunct

Several studies have confirmed that both in-person expert training and electronic training modules provide consistent outcomes for readers interpreting FBB PET scans, regardless of their level of expertise [12]. The effectiveness of the visual assessment training method has been further evaluated in real-world settings, particularly in the Neuraceq^®^ PASS 1 study (EUPAS12145) [13]. In this study, 50 nuclear medicine physicians assessed 20 FBB PET scans at baseline and re-assessed the same scans six months later. All participating physicians had received training in interpreting FBB PET images using approved educational materials and had different levels of experience in assessing amyloid PET scans. Their performance was then compared to that of 15 quantitative pipelines analyzing the same 20 FBB PET scans [15].

**Figure 5:**
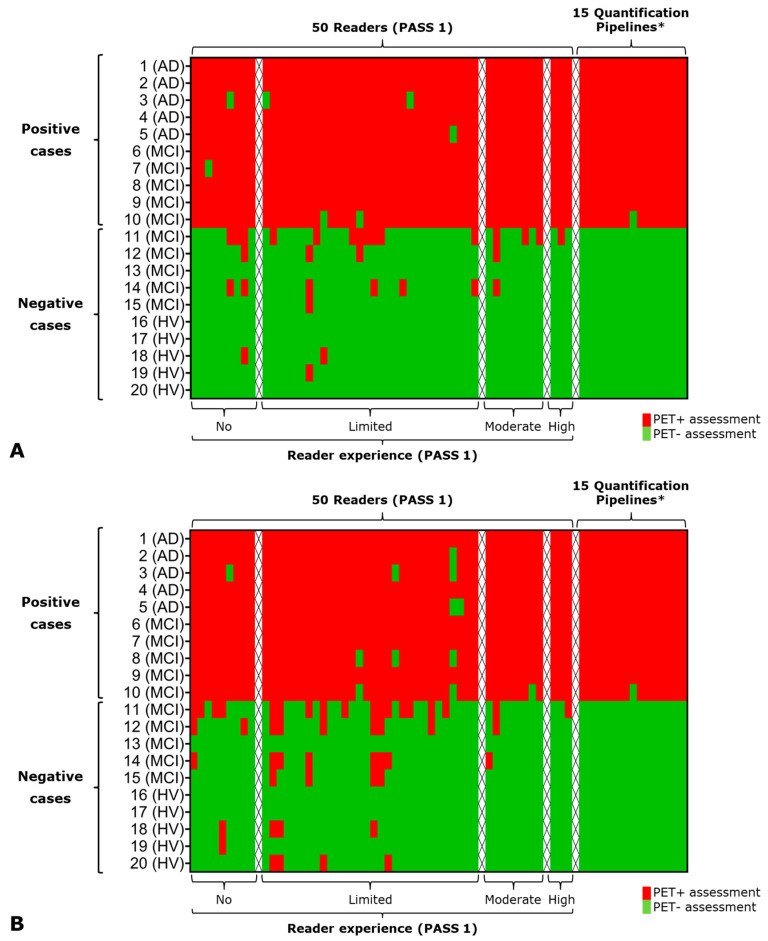
Heat maps of 50 readers (PASS 1), grouped based on experience level, and 15 quantification methods (X-axis). Scans 1–10 were classified as amyloid PET+ and scans 11–20 as amyloid PET-, based on a previous consensus read (Y-axis). PET+ and PET-assessments are shown in red and green, respectively. Experience level classification: No: 0 routine scans per month; limited: 1–5 scans per month; moderate: 6–10 scans per month; high: >25 scans per month. Panel (**A**) shows results of the baseline read and (**B**) shows the results of the follow-read of the same images 6 months later. * Quantification pipelines and quantification results as reported in [15].

At baseline assessment (see Figure 5A), readers achieved high sensitivity and specificity (>90%), independent of their experience level. Quantitative analysis across all methods also demonstrated high accuracy, with only one misclassification out of 300 assessments.

At follow-up assessment, overall sensitivity and specificity remained high (>90%). A slight increase in false-positive assessments was observed compared to baseline, resulting in a decrease in overall specificity (−3.6%). In comparison, only a marginal increase in false-negative assessments was observed, resulting in a minimal decrease in sensitivity (−1.0%). Both effects were primarily observed among readers with “no” or “limited” experience (Figure 5B), but overall, the reading method and instructions in the product characteristics [42] were still well-understood and effectively applied.

The PASS1 results indicate that reader training is effective and long-lasting, benefiting both experienced and naïve readers.

A high concordance between visual assessment and quantification has been consistently reported [15,53,54,57]. However, since amyloid accumulation is gradual [6], assessments can be more challenging in cases with intermediate amyloid levels or those near the positivity threshold [15,47]. In such instances, PET image quantification can provide valuable supplemental information to support visual assessment. A recent prospective study focusing on challenging amyloid PET cases demonstrated that quantification significantly enhanced both inter-reader agreement and confidence in the scan assessment following the disclosure of quantitative results [58].

## 4. Evolving Value of Florbetaben PET in Alzheimer’s Diagnosis and Management

The FDA and EMA approvals of florbetaben PET in 2014 were based on its diagnostic efficacy and safety; however, more widespread clinical adoption faced several barriers, such as a global lack of reimbursement. At that time, the diagnosis of AD was in a transitionary period, with limited clarity on if and how to incorporate biomarker information into the routine workflow. The diagnostic guidelines for AD remained focused on clinical symptoms [62]. While the 2013 Appropriate Use Criteria (AUC) for amyloid PET [63] provided some direction for specific clinical scenarios, the recommendations were broad and lacked detailed guidance for routine clinical application. This uncertainty was further reinforced by the absence of disease-modifying drugs (DMDs), which limited the perceived value of amyloid PET in clinical settings. In fact, the Centers for Medicare & Medicaid Services (CMS) determined in 2013 that there was insufficient evidence to support amyloid PET for diagnosing AD [64], effectively restricting its broader use in clinical practice. After initial regulatory approvals in 2014, reimbursement was lacking globally due to the perceived lack of conclusive evidence regarding its clinical utility and cost-effectiveness in routine clinical care.

However, the field has since undergone a significant transformation. Over the past decade, the diagnostic utility of amyloid PET has been validated in both specific cohorts and routine clinical settings [17,18,19]. Moreover, advances in understanding AD pathophysiology and the resulting pathological biomarkers [7,8,65], along with therapeutic innovations [33,34] have reshaped the diagnostic landscape. Current guidelines [7,8] now support biomarker-based models that define AD in vivo using biomarkers. In addition, updated AUC criteria [65] provide explicit pathways for incorporating amyloid PET into clinical practice.

In the past decade, extensive and persuasive evidence has emerged confirming that amyloid PET positively influences AD diagnosis, management, and outcomes across various clinical scenarios, even in the absence of amyloid-targeted therapies.

The IDEAS study in the US represents a landmark investigation that was designed in collaboration with the CMS to provide coverage with evidence development [18]. It was organized by the Alzheimer’s Association, conducted by the American College of Radiology, and supported by CMS and the manufacturers of all three approved amyloid PET imaging agents, i.e., florbetaben, flutemetamol and florbetapir. The primary objective of the IDEAS study was to evaluate the impact of amyloid PET on patient management during the 90-day follow-up period. The primary analysis included 11,409 subjects (FBB in 29.3%) who met the eligibility criteria and underwent a PET scan and a 90-day clinical follow-up visit. In accordance with prior research, approximately 45% of subjects with mild cognitive impairment (MCI) and 30% of subjects with dementia were Aβ-negative according to amyloid PET, indicating the absence of AD pathology [66,67]. As such, these cases were classified as having a misdiagnosis of AD. A change in overall management between pre- and post-PET visits was observed in 60.2% of MCI and 63.5% of dementia subjects after receiving information on the amyloid status [18]. To complement this, after receiving information from one amyloid PET scan, there was a change in formal diagnosis from AD to non-AD in 25% of patients and from non-AD to AD in 10.5% of patients, highlighting the strong positive and negative predictive value of amyloid PET [18].

In parallel, the Amyloid Imaging to Prevent Alzheimer’s Disease (AMYPAD) project was conducted in Europe over a period of six years. AMYPAD significantly advanced the understanding of amyloid PET’s role in AD diagnosis and treatment. The AMYPAD consortium, comprising 17 prestigious European institutions, collected over 3500 amyloid PET scans. One key study, the Diagnostic and Patient Management Study (DPMS), demonstrated that amyloid PET conducted early in the diagnostic process led to highly confident diagnoses for 40% of patients within just three months—3.5 times more than in patients without PET scans. The scans also resulted in a change of diagnosis in 44% of cases, compared to 11% in the non-PET group [68].

Meta-analyses and systematic reviews [20,69,70,71] reinforce the important role of amyloid PET in clinical practice, showing diagnostic revisions in approximately 30% of cases—consistent with the rate of clinical misdiagnosis identified through post-mortem pathology [22]. Additionally, knowledge of amyloid status influenced treatment changes in 20% to 45% of cases, underscoring the value of FBB PET in minimizing misdiagnosis, preventing inappropriate treatments, and optimizing patient care.

However, all published studies, including the large-scale multicenter studies IDEAS and AMYPAD, included selected research populations, which may not reflect real-world daily clinical practice. To address this, the Alzheimer Biomarkers in Daily Practice (ABIDE) project embedded amyloid PET into routine clinical care for unselected memory clinic patients. Even in this less diagnostically challenging memory clinic cohort, amyloid PET was associated with substantial changes in diagnosis and management [19,72]. Moreover, the ABIDE data also explored the broader impact of amyloid PET on institutionalization, mortality, and healthcare costs. The findings indicated that incorporating amyloid PET into the diagnostic process, even without DMDs, can be valuable for patients. Those who underwent amyloid PET had more favorable outcomes, including lower institutionalization and mortality rates, as well as reduced healthcare costs, suggesting that amyloid PET not only improves diagnosis but also enhances patient outcomes and reduces economic burdens [73].

Real-world evidence from the Neuraceq^®^ PASS2 (EUPAS13366) study further confirmed the successful integration of FBB PET into European clinical practice. Data from 126 clinical-routine patients showed that the usage patterns of FBB were largely consistent with the SmPC, with 97% (122/126) of patients considered to be on the label [13].

### 4.1. Florbetaben PET in Complex Clinical Situations

The clinical utility of FBB PET has been well-documented, especially in complex diagnostic cases. Ceccaldi et al. (2018), in the Neuraceq Utility Study in AD (NEUUS) in France, explored whether florbetaben PET imaging provides added value for patients with complex dementia presentations and high levels of diagnostic uncertainty in the context of the existing healthcare framework in French memory centers [17]. Amyloid PET was only introduced in a “narrow” clinical situation as a “second-line” indication for patients in whom the etiology of symptoms remained uncertain after a complete diagnostic workup and in the absence of conclusive CSF results. Ceccaldi et al. (2018) demonstrated that in patients for whom CSF analysis was indicated but either not feasible (for medical or personal reasons) or considered as non-contributory (e.g., uninterpretable due to technical issues, borderline values, partially abnormal biomarker profiles, or inconsistencies with clinical findings), FBB PET led to diagnostic changes in 66.8% of cases and improved confidence in 81.5% of patients. Furthermore, it influenced patient management in 80% of cases [17]. In a subset of patients with ambiguous CSF results (discordance of individual CSF markers), an additional evaluation with FBB PET led to significant changes in diagnostic confidence and therapeutic management [74].

Another study assessed the incremental value of amyloid PET with FBB and CSF biomarkers on AD diagnosis and confidence in suspected AD cases [75]. Using a randomized design, patients were assessed by two dementia experts over three rounds: (1) baseline diagnosis, (2) after information about either biomarker of FBB PET or CSF, and (3) after adding the second biomarker. While both biomarkers boosted diagnostic confidence, amyloid PET had a greater impact on diagnostic confidence, even when used after CSF. In contrast, CSF did not provide additional benefits when used after PET. Positive FBB PET scans solidified AD diagnoses, while negative FBB PET scans led to significant changes in diagnoses in patients. According to the raters’ feedback, the diagnostic impact of CSF was considerably lower in comparison to PET. The study concluded that amyloid PET provides greater diagnostic clarity and confidence than CSF, especially in clinically ambiguous cases [75]. Several studies have shown that amyloid PET has the greatest impact on cases that can be considered clinically challenging, as the initial diagnostic confidence is low [17,76,77].

### 4.2. Longitudinal Florbetaben PET

Longitudinal amyloid PET imaging has provided valuable insight into the progression of AD. Emerging research suggests that amyloid quantification, particularly through Centiloid scaling, can provide important information for predicting disease progression [60,61]. Two examples of clinical trials utilizing florbetaben PET in longitudinal settings are the Fundació ACE Healthy Brain Initiative (FACEHBI) and the Longitudinal Early-Onset Alzheimer’s Disease Study (LEADS).

FACEHBI is a longitudinal study of aging, cognition, lifestyle and biomarkers in 200 individuals with subjective cognitive decline (SCD), performed at Ace Alzheimer Center Barcelona since 2014, and has included multiple clinical assessments and FBB PET scans and the collection of fluid biomarkers for each participant [78]. The follow-up FBB scans in the FACEHBI study have been essential for understanding the natural accumulation of amyloid in the SCD population and how this is linked to other biomarkers and risk of cognitive decline—including conversion to MCI. A key finding from this study is that a higher amyloid burden measured by FBB PET before AD/MCI clinical symptoms appear is linked to an increased chance of cognitive decline [79].

The LEADS study aims to compare cognitive, functional, and biomarker differences between early-onset Alzheimer’s disease (EOAD) and late-onset AD (LOAD). It tracks over 500 participants, collecting MRI, amyloid and tau PET scans, cerebrospinal fluid, and genetic data. FBB PET is used in the study, amongst other tracers, to assess amyloid plaque buildup in EOAD patients, comparing their amyloid burden with that in LOAD cases to identify unique patterns of disease progression. So far, FBB PET has revealed variations in amyloid burden between early- and late-onset AD, contributing to a better understanding of disease progression in younger patients [80].

### 4.3. Florbetaben in Underrepresented Populations

The inclusion of underrepresented populations in AD research is important to understand how the disease manifests across different racial and ethnic groups. Studies using FBB PET, such as the Healthy and Aging Brain among Latino Elders (HABLE) study and the New Imaging Dementia—Evidence for Amyloid Scanning (New IDEAS) study, are expanding our knowledge of how amyloid pathology and biomarkers may differ among these populations.

The HABLE study is a longitudinal study focused on the A/T/N biomarkers within a multi-ethnic population. This study, which is the first ever performed on this scale, began in 2018 and is designed to capture various biomarker data primarily within Mexican Americans (MAs) and non-Hispanic whites (NHWs). The HABLE study is hoping to determine whether there are differences in amyloid and tau accumulation within different racial and ethnic groups throughout the course of AD using FBB and PI-2620 PET scanning (tau tracer), respectively. Beyond this, fluid biomarkers for amyloid, tau, and neurofilament light chain (NfL), MRI, neuropsychological testing, clinical labs, functional assessments, and other proteomic assays will be completed. Differences in biomarker profiles will be analyzed between the racial and ethnic groups while also seeking to determine whether fluid biomarker profiles will be adequate to screen for individuals who should receive further confirmatory diagnostics such as PET imaging. Early data from this study demonstrate differences in proteomic profiles between MA and NHW groups [81]. Differences in AT(N) timing, sequence, prevalence, and progression vary between MA and NHW [82], and there is a high degree of variability within plasma biomarkers both due to comorbidities and ethnicity [83], as well as variability in AD risk allele frequency and clinical impact between ethnic groups [84,85]. This study continues to recruit and is likely to provide substantial contributions to our understanding of the ethnic differences observed in AD.

The New IDEAS study is a large, multi-center research initiative aimed at expanding the findings from the original IDEAS study. It focuses on using amyloid PET scans to help diagnose AD in patients with mild cognitive impairment (MCI) or dementia of uncertain origin. A key goal was to determine how amyloid PET imaging impacts patient management and health outcomes, particularly in underrepresented racial and ethnic groups, such as Black and Hispanic populations. The study is no longer recruiting participants and originally aimed to enroll 7000 participants balanced across racial and ethnic groups. It was designed to address gaps in knowledge about the diagnostic value of amyloid imaging across different subpopulations and clinical presentations. FBB PET, along with other amyloid tracers, was used in the study to detect amyloid plaques in the brain, aiding in Alzheimer’s diagnosis and management decisions. Preliminary findings indicated that the inclusion of more racially and ethnically diverse participants (particularly Black and Hispanic patients) provided valuable insights into the different binding patterns of FBB PET across these populations compared to NHW [86]. Additionally, New IDEAS scientists are currently exploring differences in management and outcomes based on whether patients presented with typical or atypical Alzheimer’s symptoms, which is essential for tailoring treatment approaches and understanding the broader spectrum of AD.

### 4.4. Reimbursement Landscape

The rationale for the PET assessment of amyloid pathology has become increasingly evident, particularly in the context of amyloid-targeted therapies. The approval of amyloid-targeting disease-modifying drugs (DMDs) in 2023 and 2024, such as Leqembi^®^ [87,88] and Kisunla^TM^ [89], both of which require amyloid confirmation prior to treatment, has amplified the value of amyloid PET. These therapeutic advances have transformed amyloid PET from a purely diagnostic tool to a critical element in selecting patients for amyloid-targeting therapies. With this, the value of an earlier and more accurate AD diagnosis facilitated by amyloid PET, together with its value as an enabler of DMDs, has been recognized, leading to broader reimbursement in the US [90].

Together, these findings indicate that today there is greater clarity on when and how to incorporate amyloid PET imaging into routine care and that its incorporation brings positive value in terms of showing diagnostic utility and confidence, improving patient- and treatment-related outcomes, and potentially reducing healthcare costs. Advances in medical care, such as the IDEAS study [18] or from amyloid-lowering DMD trials for AD [33,34], have led to expanded access to amyloid PET by the CMS [90].

In Europe, the reimbursement landscape for amyloid PET has also been evolving. Spain was one of the first EU countries where a commercial amyloid PET radiopharmaceutical was reimbursed [91]. In Italy, amyloid PET reimbursement covers both the procedure and the tracer, but the real limitation lies in the hospital budget [92]. This budget constraint significantly impacts the accessibility and broader use of amyloid PET in clinical practice. Progress has also been made in countries like France and Germany despite previous challenges. The French HAS (Haute Autorité de Santé) initially issued a negative opinion on all amyloid PET tracers, deeming them to provide insufficient clinical benefit; it considered that amyloid PET had no place in the diagnostic strategy of subjects with cognitive impairment. However, the florbetaben HAS dossier was amended with the latest evidence on clinical utility and resubmitted to HAS. The re-evaluation led to a favorable opinion in 2023. A substantial clinical benefit was recognized by HAS for cases where the cause of cognitive impairment remained uncertain after physician assessment, or when CSF biomarker testing is contraindicated, impractical, or inconclusive—this is the population that was studied with FBB PET and reported by Ceccaldi et al. [17]. The favorable opinion from HAS now permits the use of commercial products in France in the clinical routine setting [93].

In Germany, amyloid PET is not generally reimbursed by statutory health insurance despite the emerging evidence from studies supporting its diagnostic value [17,18,20,69]. Different from pharmaceuticals, the German Federal Joint Committee (G-BA, Gemeinsamer Bundesausschuss, Berlin, Germany) assesses the possible reimbursement of amyloid PET radiopharmaceuticals according to their appraisal procedures for medical devices. The potential of amyloid PET was recognized by the G-BA, but a gap in evidence was identified regarding the demonstration of patient-related benefits of amyloid PET for unclear diagnoses, even after guideline-compliant assessments. To address this, the ENABLE study, a Coverage with Evidence Development (CED) study, is underway to determine whether amyloid PET improves patients’ functional outcomes, and amyloid PET is reimbursed in this study context in Germany [94].

## 5. Use of Amyloid PET in Clinical Trials for Disease-Modifying Drugs (DMDs)

Amyloid PET imaging has become a cornerstone in clinical trials for and the application of new AD therapeutics, particularly in confirming eligibility criteria (i.e., presence of amyloid plaques) and in monitoring the biological response to amyloid-lowering therapies. Also, it allows for the reliable detection of small annual amyloid changes [49,50] and has contributed significantly to our understanding of amyloid accumulation and spread in the brain in observational studies [47,95].

Recently, and for the first time, amyloid-lowering therapies have demonstrated a significant slowing of cognitive decline in AD patients [87,88,89]. While these treatments do not prevent disease progression and a cure is still not achieved, they represent a critical milestone in the treatment of AD. This advancement will further stimulate research aimed at refining the timing of therapeutic interventions along the AD continuum, optimizing treatment regimens, managing side effects, and exploring combination therapies for further improving treatment success. Amyloid PET imaging, including FBB, played a pivotal role in the development of these therapies. In these studies, large reductions in amyloid load were measured in treatment groups compared to placebo [33,34]. The changes in amyloid burden were determined using established quantitative methods, such as the Centiloid approach [33,34,96,97], or with visual assessment [98].

Here, amyloid PET has proven to be particularly valuable in confirming the presence of the therapeutic target and in tracking amyloid level over three distinct phases: (1) accumulation, (2) removal, and (3) re-accumulation of amyloid deposits, each of which has significant clinical implications for the management of AD.

### 5.1. Amyloid Accumulation

**Figure 6:**
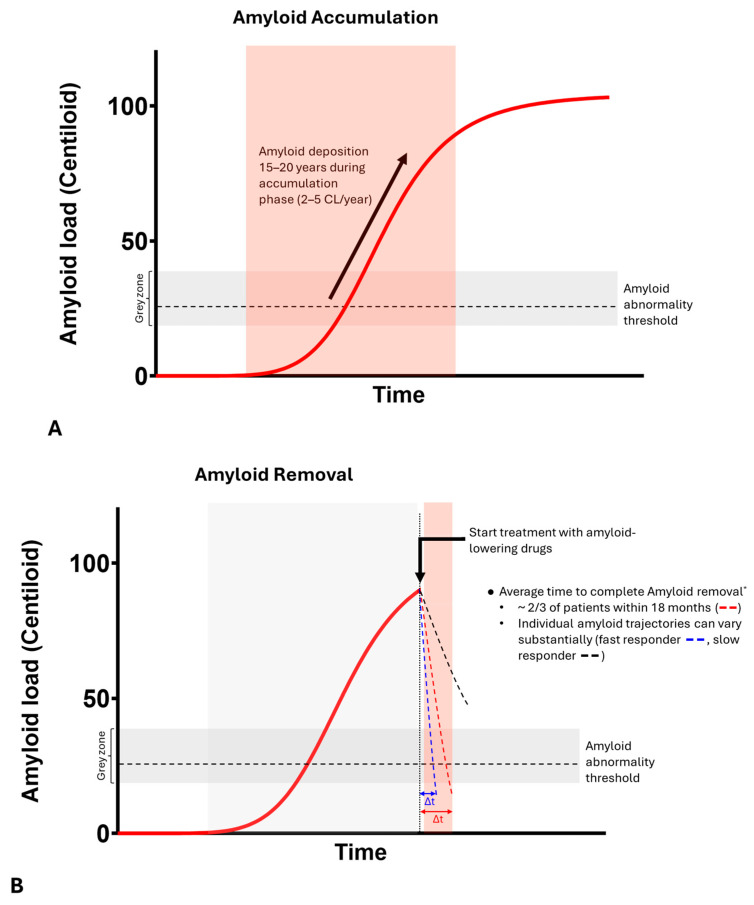

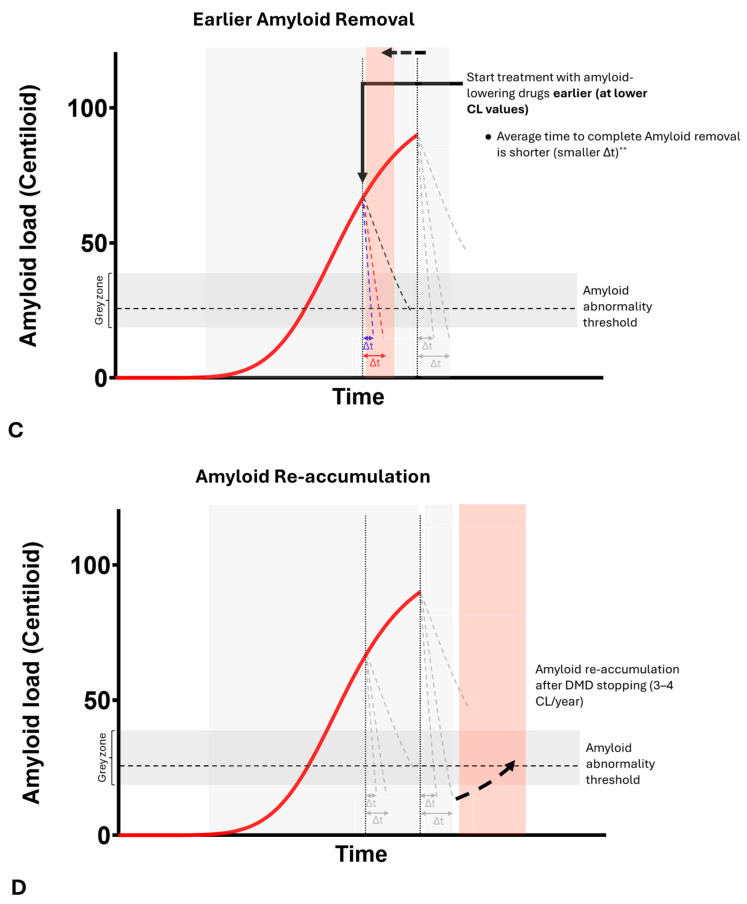
Illustration of amyloid accumulation, removal and re-accumulation. (**A**). The amyloid accumulation phase is a relatively slow process with amyloid increases in the range of 2–5 CL per year [15,95] (**B**). The rate of amyloid removal during treatment with amyloid-lowering drugs such as lecanemab and donanemab is ~20 times faster than the natural rate of amyloid accumulation [33,34] (**C**). Treatment start earlier with amyloid-lowering drugs reduces average times for complete amyloid removal [36] (**D**). Re-accumulation phases are relatively slow processes with amyloid increases in the range of 3–4 CL per year [32,99]. * as published in [34], ** as published in [36].

The amyloid accumulation phase is a relatively slow process with amyloid increases in the range of 2–5 CL per year (see Figure 6A) [15,95].

### 5.2. Amyloid Removal

In contrast, the amyloid removal phase can be much faster with amyloid-lowering drugs such as lecanemab (Leqembi^®^) and donanemab (Kisunla^TM^). These therapies demonstrate amyloid clearance rates up to 20-fold greater than natural accumulation (see Figure 6B,C; [33,34]).

Individual responses vary significantly, with approximately one-third of patients not achieving full amyloid clearance (i.e., amyloid negativity) within 18 months of treatment [33,34]. Baseline amyloid levels play a critical role in determining how quickly clearance occurs; lower initial amyloid burden is associated with faster conversion to a negative amyloid signal as levels are closer to the negativity threshold (see Figure 6C; [36]). As a result, baseline PET assessments are not only important for assessing treatment eligibility, but they also inform patient-specific follow-up scan intervals, which are critical for allowing the timely cessation of AD DMD therapy regimens, thus reducing the risk of harmful side-effects for patients as well as the treatment cost burden for health insurers and patients.

### 5.3. Amyloid Re-Accumulation

After the cessation of treatment, amyloid re-accumulation rates are comparable to natural accumulation (approximately 3–4 CL per year) (see Figure 6D; [32,99]).

**Figure 7:**
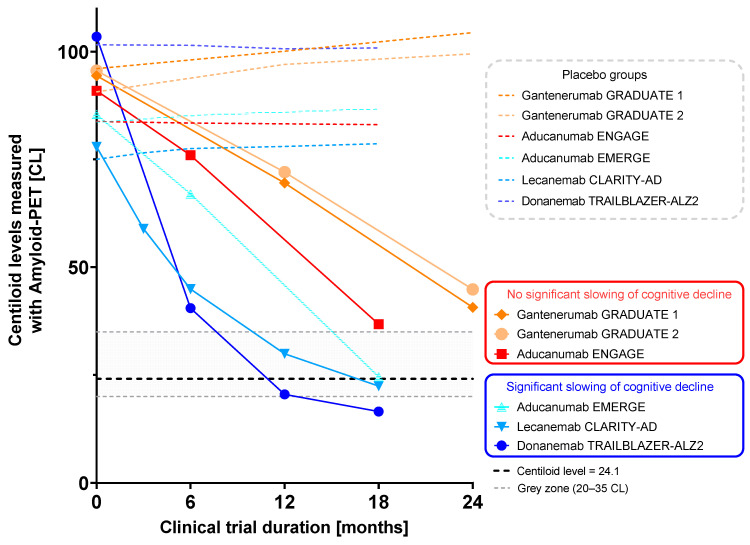
Amyloid removal profiles for Phase 3 trials of aducanumab, donanemab, gantenerumab, and lecanemab as measured by Amyloid PET using Centiloids. Sample sizes in the treatment and placebo arms varied for the individual trials and were at the last visit *N* = 614 (treatment) and *N* = 690 (placebo) for donanemab, *N* = 210 (treatment) and *N* = 205 (placebo) for lecanemab, *N* = 50 (treatment) and *N* = 41 (placebo) (Graduate 1) and *N* = 41 (treatment) and *N* = 46 (placebo) (Graduate 2) for gantenerumab, and *N* = 109 (treatment) and *N* = 109 (placebo) (Emerge high-dose) and *N* = 112 (treatment) and *N* = 124 (placebo) (Engage high-dose) for aducanumab. Data points as reported in [33,34,96,97]. Line represents 24.1 CL, the cut-off for amyloid-negativity as defined within the GRADUATE 1 and 2 trials and implemented in the head-to-head studies of donanemab vs. Aduhelm. Figure adapted from [16].

Serial amyloid PET scans were used to measure the biological response to therapy, i.e., the removal of amyloid plaques, in all recent Phase 3 trials of amyloid-lowering drugs [33,34,96,97], demonstrating the link between the amount and rate of amyloid removal and clinical efficacy [100]. In trials such as CLARITY-AD (lecanemab) and TRAILBLAZER-ALZ2 (donanemab), full clearance of amyloid within 18 months was achieved (see Figure 7) [33,34]. Both trials met their primary endpoint, showing a significant slowing of cognitive decline. Conversely, the Phase 3 studies GRADUATE 1 and 2 (gantenerumab) and ENGAGE (aducanumab) showed a slower rate of amyloid clearance, and amyloid was not fully cleared from the brain within 18 months. In these studies, no significant slowing of cognitive decline was observed [96,97].

Notably, fluid biomarkers were also used in the lecanemab and donanemab trials to monitor response to therapy, but they were unable to determine whether amyloid was fully cleared. The changes in fluid biomarkers in response to treatment were smaller compared to the direct changes in amyloid removal measured with amyloid PET imaging [101].

The clinical trials with amyloid-lowering drugs relied on the PET-based monitoring of amyloid pathology to track amyloid clearance in treatment groups and to compare with placebo groups [33,34,96,97]. PET-based amyloid load quantification, particularly using Centiloid measures, guided treatment decisions. For example, in donanemab trials, a stopping rule was applied once amyloid clearance was achieved [34], and it is recommended in the approved indication to stop dosing donanemab (Kisunla^TM^) based on the reduction of amyloid plaques to minimal levels on amyloid PET imaging [89]. This combination of baseline and follow-up PET scans has proven critical in optimizing amyloid-lowering therapy regimens and individualizing patient care. Furthermore, the future adoption of therapy monitoring in clinical practice using amyloid PET will be vital for reducing patients’ exposure to potentially harmful treatment-related side effects and also the cost burden for those reimbursing treatment. Recently, the FDA recognized amyloid clearance as a surrogate endpoint in its new guidance on developing drugs targeting early AD [102].

## 6. Summary

**Figure 8:**
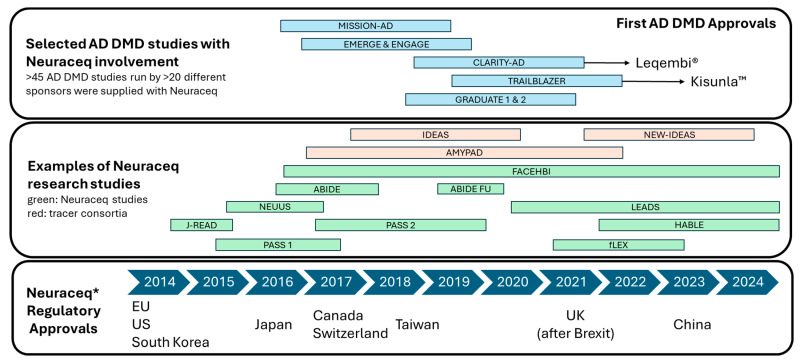
Neuraceq post approval developments. * Supplied as florbetaben in Australia, Brazil, Chile, New Zealand, The Phillipines and Thailand according to local regulations and/or exemptions to the requirement for marketing authorizations.

Figure 8 summarizes the Neuraceq developments since the first marketing authorizations were obtained in 2014. Additional regulatory approvals followed in various countries through license partners, and the product is now globally available for routine clinical use and/or research. Individual research studies are shown as examples and have been discussed (see Section 4). Here, the investigations focused mainly on demonstrations of the clinical benefits and confirming the safety in the real-world setting. In parallel, pharmaceutical companies used amyloid PET in their AD drug development trials. A few key clinical studies highlight florbetaben’s pivotal role in confirming amyloid status, significantly contributing to the successful approval of the first AD DMDs.

## 7. Outlook

### 7.1. Florbetaben PET in DMD Trials and in Routine AD Diagnostic Workup

Since its approval in 2014, florbetaben PET has evolved from a diagnostic tool aimed at excluding amyloid pathology to a pivotal component in AD drug development trials. In addition, it now plays an essential role in selecting individuals for approved amyloid-targeted therapies like Leqembi^®^ and Kisunla^TM^ [87,88,89]. Beyond its established diagnostic utility, florbetaben PET is invaluable in directly measuring the biological responses of these newly approved DMDs, where a reduction in amyloid PET signal is reasonably likely to predict clinical benefit, as recognized by regulatory agencies like the FDA [103]. This shift marks the transformation of amyloid PET from a purely diagnostic tool to an integral part of treatment selection and monitoring.

The development of the Centiloid scale as a common read-out of amyloid burden further strengthens its role, providing a standardized and tracer-independent metric for amyloid plaque quantification and facilitating patient selection in clinical trials [104]. EMA recently issued a qualification opinion endorsing the Centiloid metric as a validated measure of global amyloid load for clinical trials when combined with appropriate quality control [52]. Specific Centiloid cut-offs are now routinely used to enrich trials with participants likely to progress on key outcomes such as amyloid burden and cognition [105]. Trials like AHEAD A3 and AHEAD 45 have leveraged the Centiloid scale to select patients who are more likely to show measurable changes during the course of the study [105].

Looking to the future, the role of amyloid PET will likely expand further as new anti-amyloid therapies are developed. While first-generation amyloid-targeting immunotherapies are now approved, their limited ability to cross the blood–brain barrier and the associated safety risks of amyloid-related imaging abnormalities (ARIA) highlight the need for improved treatment options. Novel delivery systems, which allow more efficient blood–brain barrier crossing, are being explored to enhance drug penetration into the brain while minimizing adverse events like ARIA [106,107,108]. Trontinemab, a new antibody developed by Roche using the brain-shuttle mechanism, has demonstrated improved brain exposure, essentially removing amyloid plaque from the brain within three months, with no ARIA observed in the limited number of participants treated with the antibody so far [106]. With these advancements, amyloid PET will likely continue to be a cornerstone in both drug development and the clinical management of AD.

The importance of amyloid PET is, however, not solely tied to the availability of treatment options. Patients and families request accurate information about the causes of cognitive impairment and the associated clinical prognosis [109]. Amyloid PET is already instrumental in informing patients and their families about the underlying causes of cognitive impairments [110,111] and in shaping their understanding of prognosis [41]. Predictive models or risk calculators have been developed based on the well-characterized accumulation patterns of amyloid, helping to estimate the time course leading to symptomatic AD [109]. An accurate diagnosis is also the gateway to proper care, as demonstrated by many studies emphasizing the significant clinical utility of amyloid PET [18,69]. Moreover, patients who undergo amyloid PET during the diagnostic workup experience fewer hospital admissions, lower rates of institutionalization, and reduced healthcare costs in the years following diagnosis [73]. Thus, the value of amyloid PET extends far beyond treatment; it provides critical insights for diagnosis and prognosis, both of which are essential for guiding care strategies for patients living with or at risk for dementia. To fully realize the potential of recent advancements in the field of dementia, it is crucial to emphasize the importance of timely diagnosis and accurate prognosis, in addition to maintaining a strong focus on treatment.

### 7.2. Integration with Fluid Biomarkers

Amyloid PET has become integral to the diagnostic workup of AD and is deemed appropriate for a variety of clinical scenarios [65]. Alongside imaging, fluid biomarkers such as CSF amyloid biomarkers are increasingly being used in clinical practice to confirm amyloid pathology, particularly in specialized settings. CSF testing, while valuable for detecting amyloid pathology, requires LP, which is an invasive procedure that many patients prefer to avoid in favor of less invasive imaging tests [112]. Additionally, some patients may be contraindicated for LP, further limiting its accessibility [113]. Even when performed, CSF testing is not without challenges, and results can be ambiguous, inconsistent with clinical information or uninterpretable [17,114].

Blood-based biomarkers, meanwhile, are gaining traction as screening tools for AD, potentially enabling earlier diagnosis and more efficient referrals to specialist care. While blood biomarkers hold significant promise for screening purposes and streamlining diagnostics, challenges remain regarding their implementation across diverse populations and ensuring diagnostic accuracy [31]. Importantly, neither CSF nor blood biomarkers are currently suitable to determine when amyloid is fully cleared from the brain to stop treatment, maintaining the critical role of amyloid PET.

### 7.3. Amyloid PET and Tau PET

While amyloid PET has taken center stage in the context of amyloid-targeted therapies, tau PET is increasingly recognized for its complementary role. Amyloid PET is highly sensitive in detecting early AD pathology, making it indispensable for early diagnosis and intervention [87,88,89]. In contrast, tau PET, which detects neurofibrillary tangles, is emerging as a valuable tool for staging disease progression, as tau pathology correlates more closely with cognitive decline. Some clinical trials have already categorized individuals into low- and high-tau PET groups based on the assumption that participants with low-tau PET would benefit the most from amyloid-lowering drugs [34]. Moving forward, the integration of amyloid and tau PET imaging is expected to offer a more comprehensive view of Alzheimer’s pathology, refining both diagnostic and prognostic capabilities. This combined approach could also enhance personalized treatment strategies as more DMDs are approved and introduced into clinical practice.

## 8. Conclusions

Following the successful completion of the FBB clinical development program and the attainment of marketing authorizations, a period of real-world experience ensued, during which its clinical utility had to be demonstrated to overcome barriers for broader adoption. This was accomplished in parallel with the evolving AD diagnostic landscape. Through large consortia trials, the clinical utility of amyloid PET was collectively validated by the community. With this foundation and the availability of first effective AD DMDs, a new era has begun. The full potential of amyloid PET can now be realized to benefit patients, expand our understanding of the disease, and ultimately pave the way for future therapies that may halt disease progression.
